# Massive Hemothorax Caused by Removal of Percutaneous Transhepatic Abscess Drainage Tube for Bile Leak After Subtotal Cholecystectomy: A Case Report

**DOI:** 10.7759/cureus.43310

**Published:** 2023-08-10

**Authors:** Yuki Hoshi, Satoru Ishii, Tsukasa Takizawa, Hikaru Tamura

**Affiliations:** 1 Surgery, Nasu Red Cross Hospital, Otawara, JPN

**Keywords:** subtotal cholecystectomy, percutaneous transhepatic abscess drainage, hemothorax, chronic cholecystitis, bile leak

## Abstract

A 59-year-old man with a past medical history of gallstones was diagnosed with acute cholecystitis and received antibiotic treatment. He was discharged after ten days of hospitalization and was due to undergo laparoscopic cholecystectomy. Three months later, however, he had to be readmitted due to a recurrence of acute cholecystitis. Subsequently, laparoscopic reconstituting subtotal cholecystectomy was performed because Inflammation of the gallbladder was severe. At the first postoperative outpatient visit, the patient reported obstructive jaundice, and computed tomography (CT) scan revealed fluid collection in the hepatic bed and a missed common bile duct stone. Percutaneous transhepatic abscess drainage (PTAD) was performed on admission, and endoscopic stone removal was attempted the following day but was challenging due to a periampullary diverticulum. During laparotomy for stone extraction, the patient experienced prolonged shock and CT showed bleeding from the liver and massive right hemothorax. After open chest drainage and hemostasis, transcatheter arterial embolization (TAE) was performed. Such a case has never been reported before, and the PTAD tube should be handled cautiously.

## Introduction

Percutaneous transhepatic abscess drainage (PTAD) is a safe and effective drainage method for hepatobiliary infections [1]. It is used mainly for liver abscesses and abscesses in the hepatic bed. The technique involves first puncturing the abscess in the supine or left lateral decubitus position, using ultrasound to puncture the abscess from the right intercostal space through the liver parenchyma, avoiding blood vessels and intrahepatic bile ducts [2]. A drainage tube (often a pigtail catheter) is placed into the abscess using the Seldinger technique under fluoroscopy [2]. Bleeding is a common complication of PTAD, mostly occurring at insertion [3]. Bleeding at removal is rare [4], and hemothorax due to bleeding at removal has not been reported. Here, we present a case of massive hemothorax due to removing the PTAD tube after laparoscopic subtotal cholecystectomy.

## Case presentation

A 59-year-old man with a history of type 2 diabetes, dyslipidemia, and hypertension presented to the outpatient clinic with abdominal pain. He was diagnosed with cholelithiasis with biliary colic, and the plan was to follow up on an outpatient basis because he was busy with work and did not wish to have the surgery at the time. Two days later, he visited the emergency department due to fever, chills, and abdominal pain. The patient's general condition was good, but the temperature was 38.1 °C. Blood work showed white blood cell (WBC) count of 11500/µL, C reactive protein (CRP) level of 0.48 mg/dL, total bilirubin of 2.3 mg/dL, direct bilirubin of 1.3 mg/dL, aspartate aminotransferase (AST) of 710 IU/L, alanine transaminase (ALT) of 545 IU/L, alkaline phosphatase (ALP) of 459 IU/L, gamma-glutamyl transpeptidase (GGTP) of 780 IU/L, lactate dehydrogenase (LDH) of 711 IU/L. The patient was hospitalized, diagnosed with acute cholecystitis, and discharged after ten days of antimicrobial treatment with piperacillin/tazobactam. He was going to undergo laparoscopic cholecystectomy. Three months later, however, he was readmitted due to a recurrence of acute cholecystitis.

**Figure 1:**
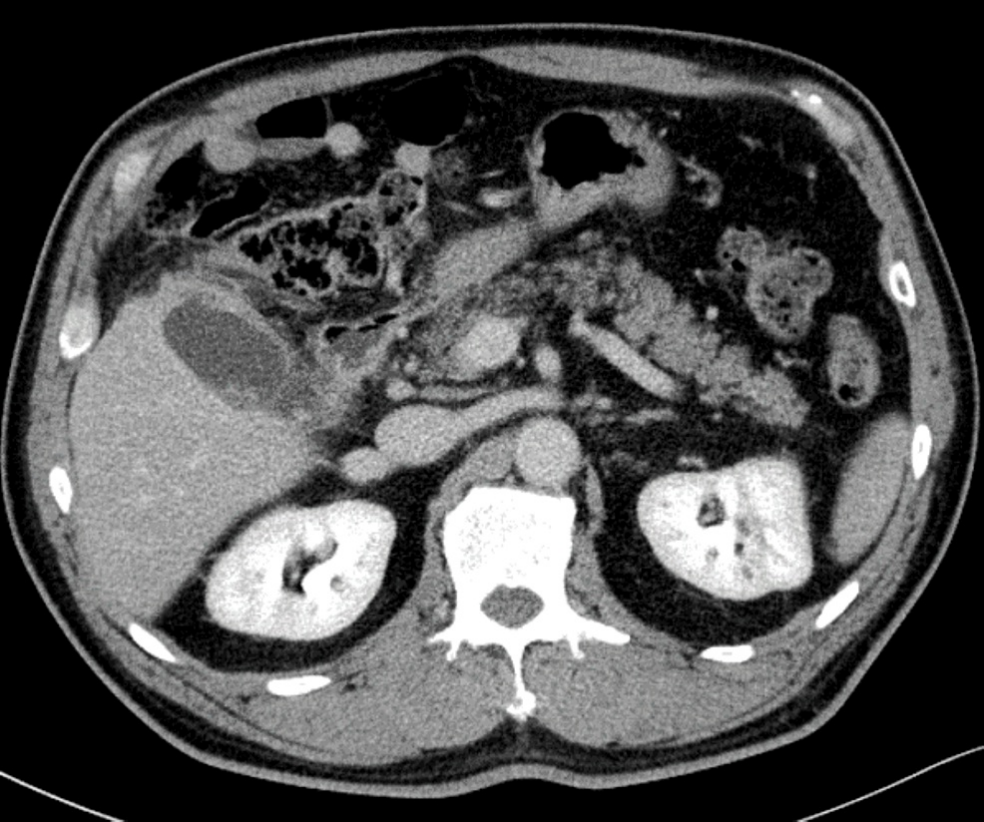
Abdominal CT at admission showed four 6-mm stones in the gallbladder, one of which was fitted into the cystic duct. The gallbladder was enlarged with abscess formation.

On admission, the patient's BMI was 26.7 kg/m^2^. He had clear consciousness, a temperature of 36.9 °C, a pulse of 81 beats/min, blood pressure of 136/77 mmHg, a respiratory rate of 20 breaths/min, and SpO_2_ of 98% (room air). The abdomen was flat and soft, with tenderness in the right upper quadrant. Blood tests revealed WBC count of 11700/µL, CRP level of 5.85 mg/dL, total bilirubin of 0.8 mg/dL, AST of 25 IU/L, ALT of 39 IU/L, ALP of 93 IU/L, GGTP of 60 IU/L, LDH of 208 IU/L, prothrombin time (PT) of 14.4 seconds, PT activity of 69.0%, prothrombin time-international normalized ratio (PT-INR) of 1.21, and activated partial thromboplastin time (APTT) of 35.1 seconds. Abdominal contrast-enhanced computed tomography (CT) showed four 6 mm stones with mild calcification in the gallbladder, one of which was fitted into the cystic duct. The gallbladder was enlarged to about 8 cm in size, and an abscess was formed around the fundus of the gallbladder (Figure 1).

**Figure 2:**
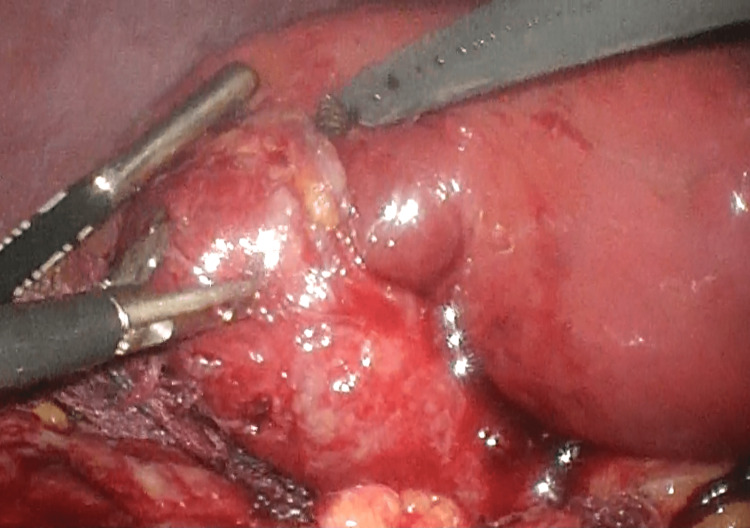
The gallbladder was hard and distended with fibrosis due to inflammation, suggesting acute exacerbation of chronic cholecystitis.

The patient was diagnosed with moderate acute cholecystitis, and laparoscopic cholecystectomy was performed. During surgery, extensive inflammatory adhesions were noted around the gallbladder to the omentum, transverse colon, and duodenum. The gallbladder was hard and distended with fibrosis due to inflammation, suggesting acute exacerbation of chronic cholecystitis (Figure 2). Since it was difficult to identify the junction of the cystic and common hepatic duct, we dissected the fundus first. The gallbladder lumen was partially opened during the dissection, and the gallbladder neck was dissected while confirming the lumen. The neck was closed with interrupted sutures of 3-0 polypropylene sutures (Prolene, Johnson & Johnson, Somerville, NJ), and subtotal cholecystectomy was performed. Finally, a negative-pressure suction drain was placed on the subhepatic surface. We considered performing cholangioscopy to confirm the absence of stones in the common bile duct. Still, we did not do so because of the high risk of common bile duct injury, the difficulty of performing cholangioscopy in a patient with subtotal cholecystectomy, and the fact that if stones were present, the patient would have required laparotomy for stone extraction, which he did not wish to undergo, preferring to perform endoscopic retrograde cholangiopancreatography (ERCP) after the surgery. The patient received ceftriaxone postoperatively and resumed eating the next day without gastrointestinal symptoms. The subhepatic drainage was serous and small during the postoperative period, so the drain was removed on the third day. The patient was discharged on the fourth day and treated with levofloxacin for one week after discharge. 

**Figure 3:**
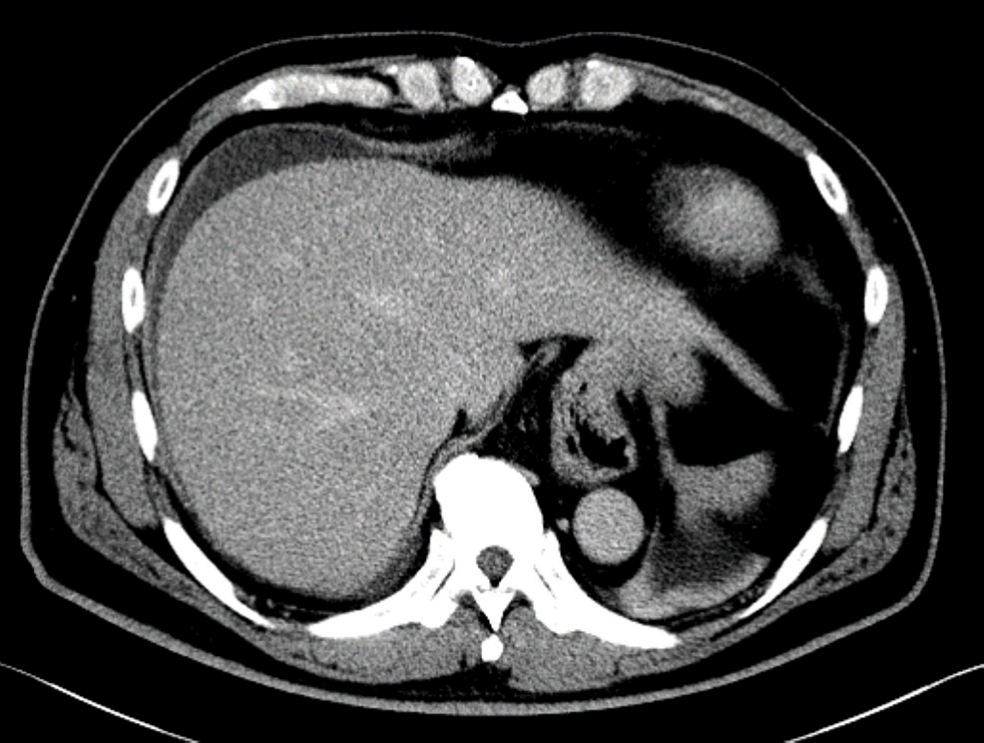
Abdominal CT at readmission showed fluid collection under the right diaphragm.

On the evening of the day of discharge, he presented to the emergency department with abdominal pain. He had tenderness in the right quadrant with radiating pain to the right shoulder, and a low-grade fever of 37.2°C was noted. Blood tests showed WBC of 10,600/µL, CRP of 9.50 mg/dL, total bilirubin of 0.6 mg/dL, AST of 33 IU/L, ALT of 51 IU/L, ALP of 122 IU/L, GGTP of 99 IU/L, LDH of 180 IU/L. CT showed fluid collection under the right diaphragm (Figure 3). A diagnosis of postoperative intra-abdominal abscess was made. Conservative treatment with antimicrobial agents was initiated, which improved symptoms and inflammatory findings. The patient was discharged on the 11th day after the surgery.

**Figure 4:**
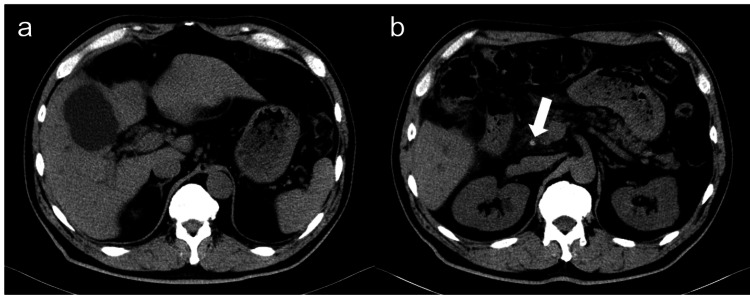
Abdominal CT at the first outpatient visit after discharge revealed (a) fluid retention consistent with gallbladder bed and (b) calcified stones (white arrow) in the intra-pancreatic bile duct.

The patient reported white stools and brown urine during the first outpatient visit after discharge. Blood tests showed elevated bilirubin and hepatobiliary enzymes with a total bilirubin of 3.2 mg/dL, direct bilirubin of 2.3 mg/dL, AST of 117 U/L, ALT of U/L, ALP of 633 U/L, and GGTP of 434 U/L. CT scan revealed fluid retention in the gallbladder bed and a calcified stone in the intra-pancreatic bile duct (Figure 4). We diagnosed the patient with bile leakage caused by a stone falling from the gallbladder neck and increased pressure in the bile duct.

**Figure 5:**
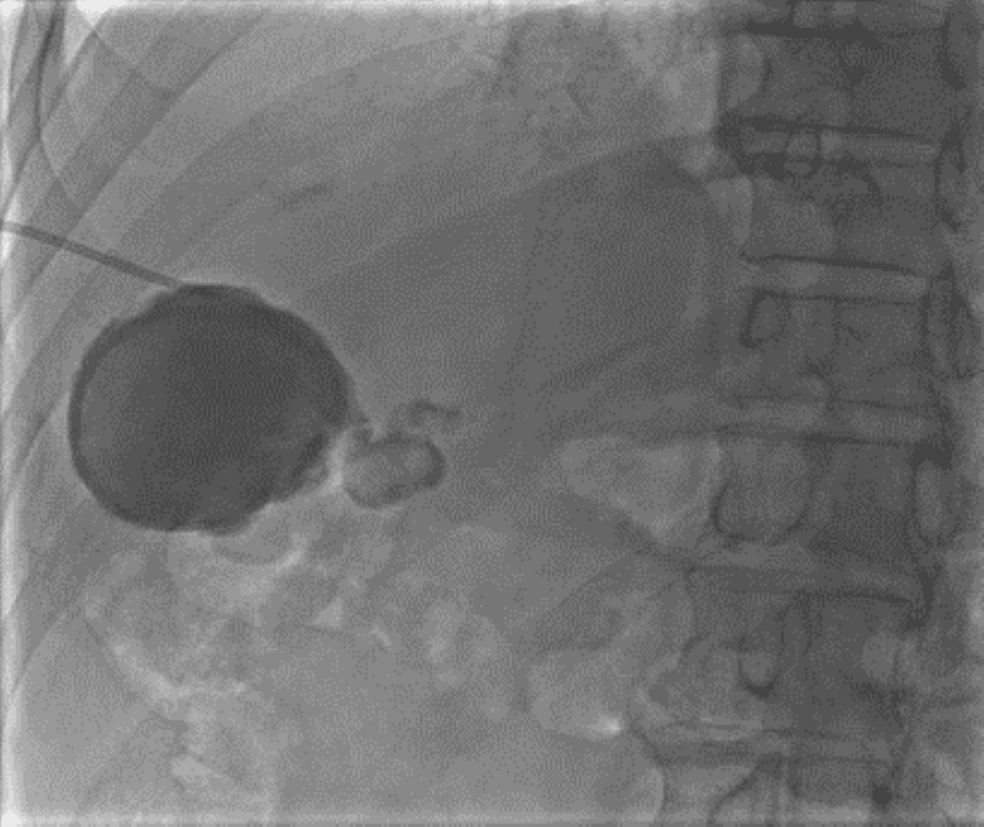
Percutaneous transhepatic abscess drainage (PTAD) finding showed contrast accumulation in the hepatic bed and dilated cystic duct.

**Figure 6:**
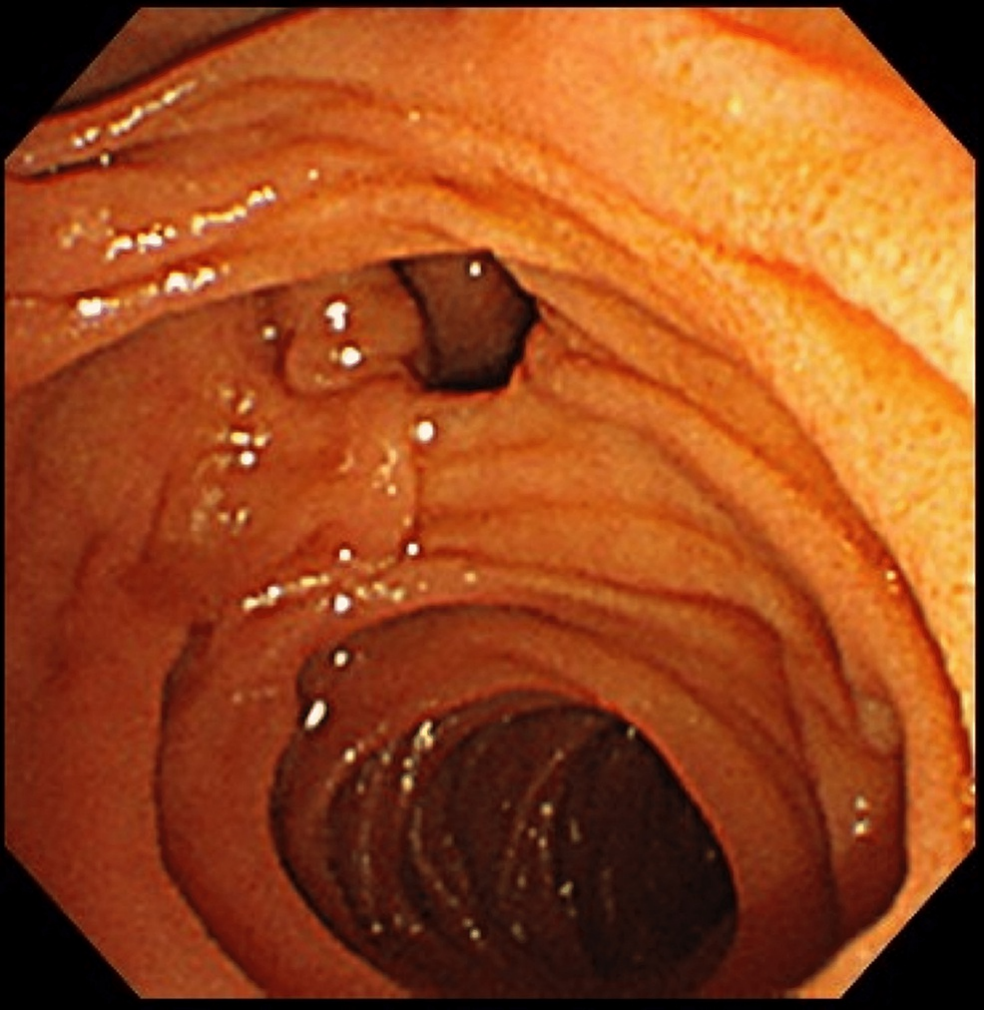
Endoscopic retrograde cholangiopancreatography (ERCP) finding - cannulation was difficult because of a periampullary diverticulum.

Upon admission to the hospital, the patient underwent PTAD (7 Fr pigtail catheter, Hanaco Medical, Saitama, Japan) to address the bile leak in the gallbladder bed (Figure 5). The following day, an attempt was made to extract the stone through ERCP. However, cannulation proved challenging due to a periampullary diverticulum (Figure 6). As a result, the decision was made to defer the stone extraction and perform surgery.

On the 27th day post the initial surgery, the patient underwent laparotomy to remove the residual gallbladder and common bile duct stone altogether. Due to the previous surgery, the residual gallbladder and the common bile duct had firmly adhered. The residual gallbladder was carefully dissected, and the drainage tube was manually removed from the body surface. The common bile duct was incised and exposed, and cholangiography was performed to extract the common bile duct stone. After verifying by cholangioscopy that there was no residual stone, the residual gallbladder was resected, and the common bile duct was sutured closed.

The patient's heart rate increased to 140 bpm during the operation, and systolic blood pressure decreased to 70 mmHg. No pressure increase was obtained despite noradrenaline at a 0.1 mg/kg/min dose. Intra-abdominal bleeding was the primary concern, but there was no bleeding in the operative field that could have caused hypotension. Hemoglobin was 10.2 g/dL, and hematocrit was 30.4%, which, together with the intraoperative findings, did not suggest massive bleeding. Anaphylaxis to intraoperative antibiotics (cefmetazole) was suspected of causing this event, although the patient did not present any other symptoms, such as skin redness. Adrenaline 0.1 mg was administered, and 1000 ml of crystalloid fluid and 250 ml of 5% albumin were fully opened. His systolic blood pressure stabilized at 100 mmHg, and noradrenaline was stopped. However, within one hour of admission to the intensive care unit (ICU), the patient's blood pressure dropped to 70 mmHg, and noradrenaline had to be re-administered at a dose of 0.1 mg/kg/min.

Also, postoperative blood sampling revealed anemia with Hb 6.2 g/dL, and four red blood cell (RBC) transfusion units were started. Despite these interventions, the patient still could not maintain his circulation, and the dose of noradrenaline was increased to 0.14 mg/kg/min. The patient was intubated and maintained oxygen saturation around 95% but continued tachypnea, with a respiratory rate of approximately 50 breaths per minute.

**Figure 7:**
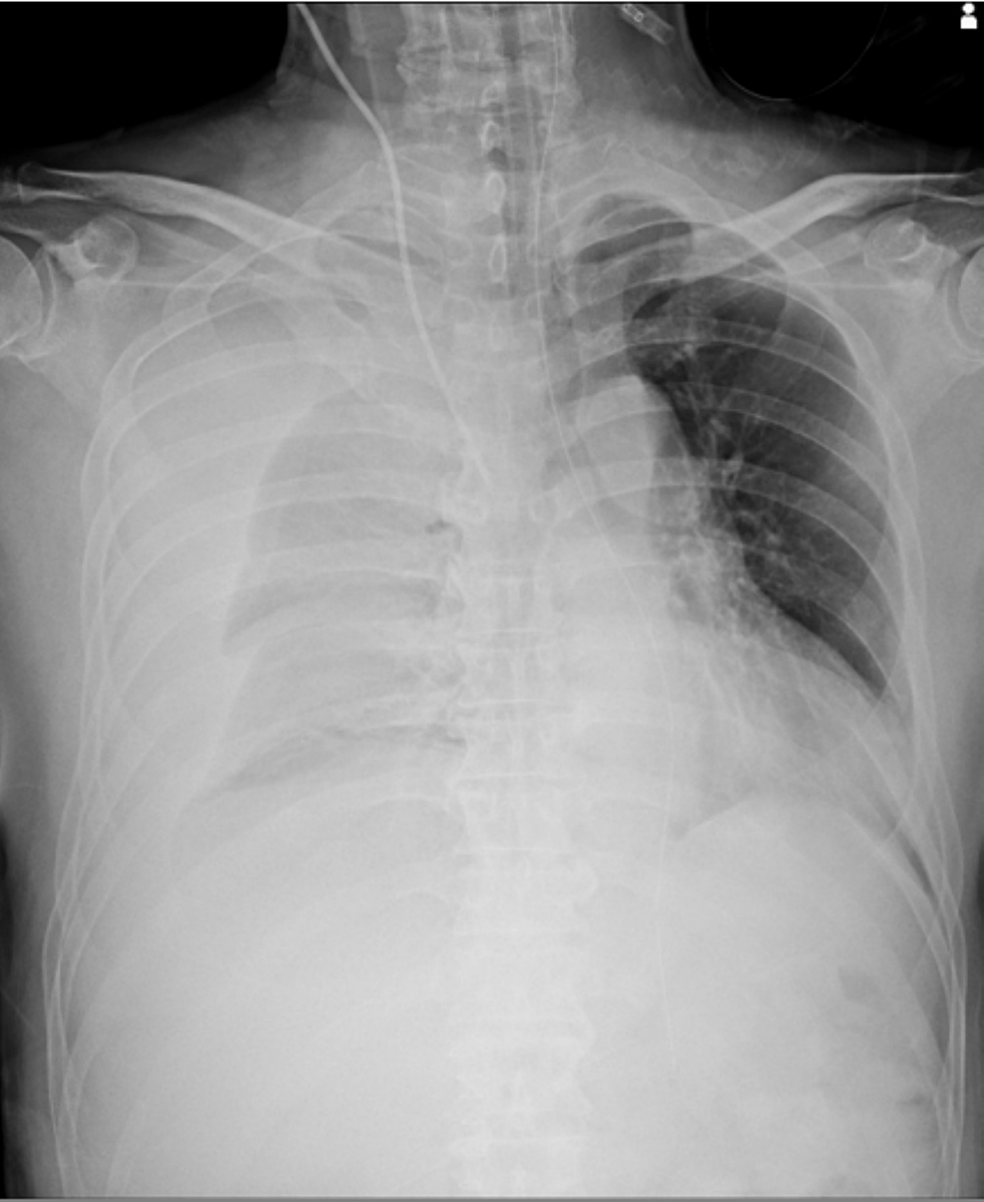
Chest radiograph after inserting a central venous catheter showed a significant right-sided consolidation, a collapsed right lung, and tracheal deviation to the left side.

**Figure 8:**
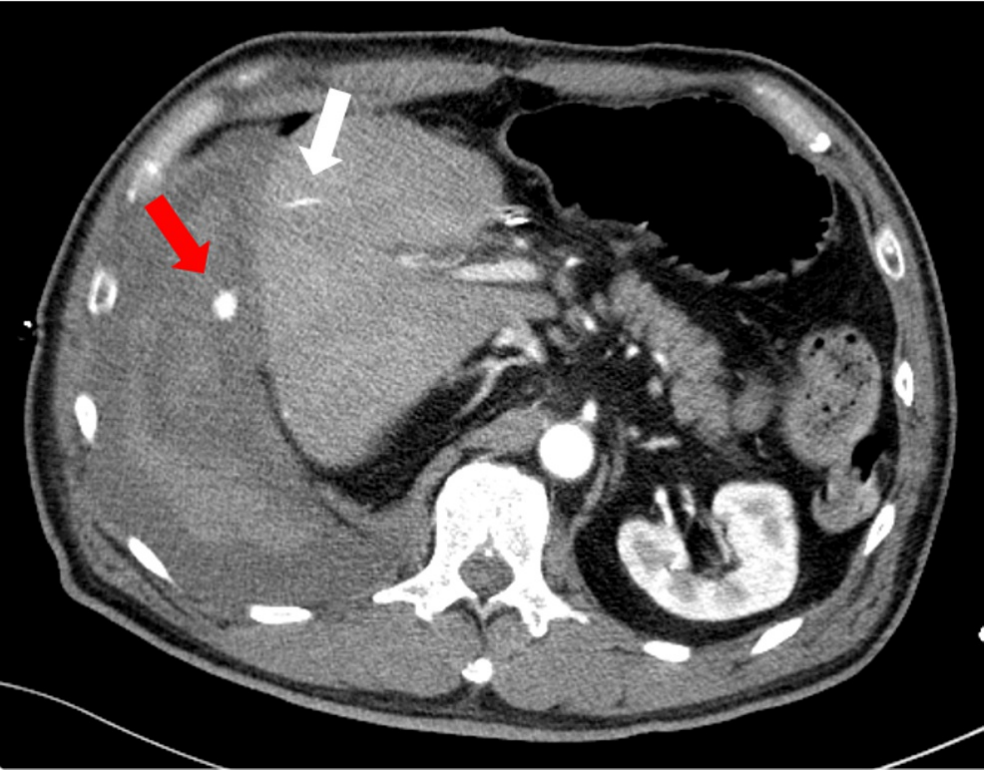
Contrast-enhanced CT of massive hemothorax showed a bleeding point in the right thoracic cavity (red arrow) and a high-density streak in the liver, suggesting hemorrhage (white arrow).

A central venous catheter was inserted through the right internal jugular vein, and a chest radiograph was taken to confirm the catheter tip position. The radiograph revealed a significant right-sided consolidation, a collapsed right lung, and tracheal deviation to the left side (Figure 7). A 20 Fr drain was inserted into the right thoracic cavity, and a large amount of bloody pleural fluid was found. A CT scan was performed to confirm the site of bleeding. A hemorrhage point was observed in the right pleural cavity (Figure 8). It was discovered that the drainage tube had penetrated the thoracic cavity, and upon removal, bleeding from the liver or diaphragm accumulated in the thoracic cavity, causing tension hemothorax. The patient was administered 12 units of RBC, 12 units of fresh frozen plasma (FFP), and 1000 mg of tranexamic acid six hours after admission to the ICU. Still, the systolic blood pressure was persistently in the 70-80 mmHg range, and the patient remained in prolonged shock. Maintaining the patient's circulatory control was challenging.

Considering the patient's unstable circulation and the difficulty of endovascular treatment at night, it was decided to perform open chest hemostasis before daybreak to address the bleeding. Under general anesthesia, the patient was placed in a left semi-lateral decubitus position with one lung ventilation. A 5 cm incision was made along the intercostal space, centered on the drain removal site. Upon opening the right pleural cavity, a lot of bloody pleural fluid and clots were found. The pleural fluid and clots were removed as much as possible, and the right diaphragm was checked, revealing bleeding that was sutured and stopped under direct vision. The right thoracic cavity was washed, and nearly all clots were removed. A negative-pressure suction drain was placed in the right thoracic cavity to complete the operation, which lasted for two hours, with a blood loss of 5007 ml. Intraoperatively, eight units of RBC and eight units of FFP were transfused.

**Figure 9:**
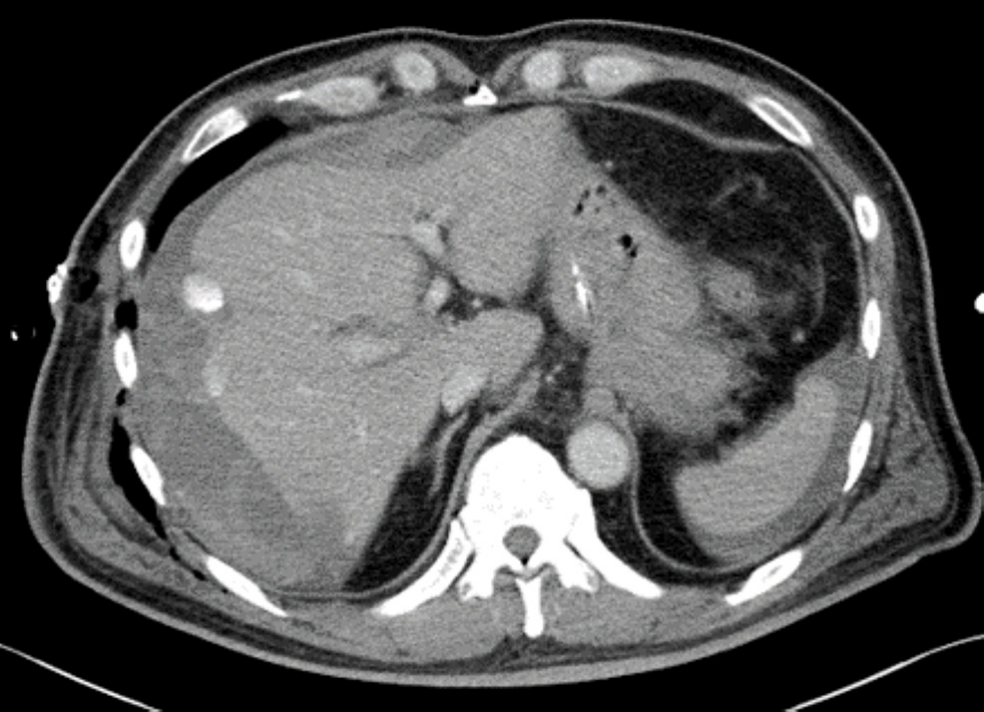
Contrast-enhanced CT after open chest hemostasis revealed bleeding from the liver into the abdominal cavity.

**Figure 10:**
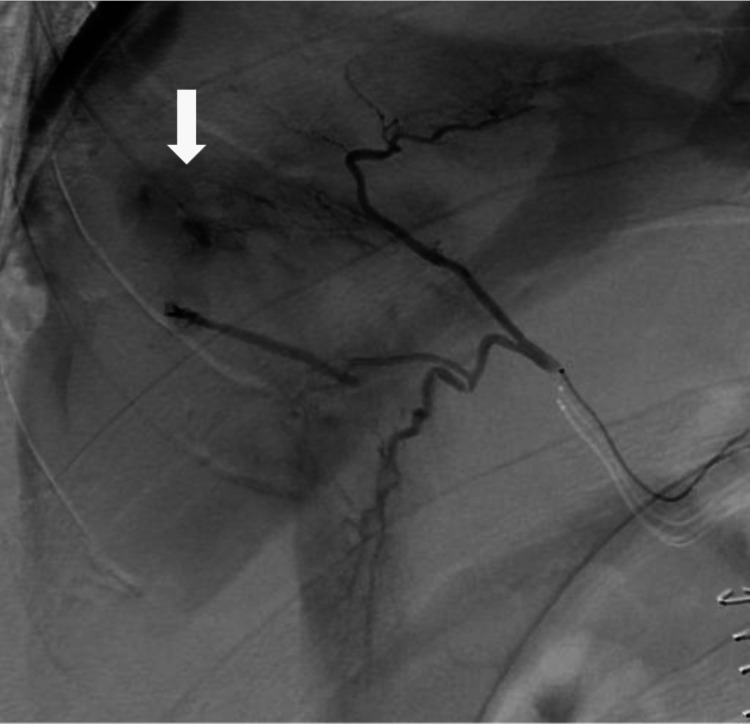
Celiac artery angiography revealed hemorrhage from the anterior segment of the right hepatic lobe.

Upon returning to the ICU, the patient's systolic blood pressure remained stable within 90-100 mmHg; however, it dropped to 60 mmHg after an hour. Despite arterial blood gas showing no worsening of anemia with Hb 8.6 g/dL, another blood transfusion was required to maintain the patient's blood pressure. In the early morning, a contrast-enhanced CT scan revealed bleeding from the liver into the abdominal cavity (Figure 9). As endovascular treatment was deemed urgent, transcatheter arterial embolization (TAE) was performed to treat the bleeding. An arterial branch of the anterior segment of the right hepatic lobe was embolized with a gelatin sponge and coil (Figure 10). Following the TAE, the patient's systolic blood pressure quickly recovered to 110-130 mmHg.

The patient was successfully extubated three days after the TAE and was transferred out of the ICU the following day. The patient continued to recover without complications and was discharged on the 13th day post-TAE. Currently, the patient is being managed as an outpatient and progressing without issues.

## Discussion

This case report describes a patient who underwent PTAD placement for biliary leakage following subtotal cholecystectomy. During the procedure, the liver parenchyma was inadvertently injured when the tube was removed intraoperatively, leading to a tension hemothorax due to the tube passing through the thoracic cavity. Although hemostasis was achieved initially through manipulation from the thoracic cavity, the fistula was subsequently ruptured, resulting in bleeding into the abdominal cavity. To our knowledge, such a case has not been previously reported. This case highlights the need for careful attention and management during cholecystitis treatment.

Laparoscopic cholecystectomy is a commonly performed surgery for cholecystitis. However, the difficulty level varies significantly depending on the degree of inflammation, fibrosis, and adhesion to the surrounding tissues [5]. In acute exacerbations of chronic cholecystitis, such as the case presented here, identifying the appropriate dissection layer in the Calot's triangle and gallbladder bed can be challenging due to the acute inflammation combined with the high fibrosis and scarring of the gallbladder wall and surrounding adhesions caused by chronic inflammation. In such cases, biliary injury is a crucial complication to avoid.

The Tokyo Guidelines 2018 suggest three bailout procedures when laparoscopic cholecystectomy is difficult: subtotal cholecystectomy, open conversion, and fundus first technique [6]. The subtotal cholecystectomy was performed laparoscopically using the fundus first technique in the present case. Acar et al. reported that intraoperative biliary injury could be avoided in almost all cases by subtotal cholecystectomy, and other postoperative complications were also investigated [7]. The results showed no cases of intraoperative biliary injury, but 21.1% of bile leaks, 14% of surgical site infection, and 10.5% of residual abscesses were postoperative complications [7]. In a systematic review by Henneman et al., the efficacy and safety of laparoscopic subtotal cholecystectomy were evaluated in a total of 625 patients in 15 articles, and bile leaks were the most common postoperative complication, which resolved spontaneously in an average of 9.5 days [8]. In a meta-analysis by Elshaer et al., they compared the postoperative complications of subtotal cholecystectomy by surgical technique [9]. There was no significant difference in the frequency of complications between removal and non-removal of the posterior wall of the gallbladder or closure and nonclosure of the gallbladder stump. Compared to laparoscopic and open surgery, laparoscopic surgery had fewer complications overall, but only bile leaks were significantly more common with laparoscopic surgery [9].

Various methods have been reported to prevent bile leakage during laparoscopic subtotal cholecystectomy [10-12]. Fujiwara et al. described a technique where the neck of the gallbladder was closed using continuous sutures with a barbed suture [10]. Kato et al. reported a case in which omentum was sutured to the gallbladder stump to prevent bile leakage [11]. Matsui et al. proposed a method where omentum was used to fill the gallbladder duct orifice via the gallbladder lumen [12].

The treatment of bile leakage after total or subtotal cholecystectomy is determined based on a comprehensive evaluation of the degree and location of bile leakage and a patient's condition. Drainage should be considered for cases not treated with conservative treatment, such as when bile leakage is extensive, or an abscess is observed [13]. The first method of drainage is often endoscopic decompression, which includes endoscopic sphincterotomy (EST), endoscopic biliary stenting (EBS), and endoscopic nasobiliary drainage (ENBD) [14]. In the present case, the gallbladder neck was considered to have ruptured due to increased intraductal pressure caused by gallbladder stones, resulting in fluid retention in the hepatic bed, which could not be drained by endoscopic procedures alone and required percutaneous transhepatic drainage. However, endoscopic cholangiography was difficult due to a periampullary diverticulum. Therefore, the only way to treat the bile leak was to remove the fitted stone by surgery. It was possible that cholangiography at the time of subtotal cholecystectomy to check for residual stones could have prevented postoperative bile leakage.

One of the complications of percutaneous transhepatic drainage, such as PTGBD and PTAD, is bleeding [3]. Bleeding is mainly caused by damage to the hepatic artery, portal vein, or intercostal artery during puncture [3], and bleeding after removal is rare [4]. In the present case, there was no significant change after placement of the drainage tube, and massive bleeding from the liver occurred after removal, suggesting that blunt hepatic injury was caused by a curved tip of the pigtail catheter during removal. Although our case is the only case of massive hemothorax through a fistula caused by a transthoracic puncture, there are a few reports of hepatic injury caused by removing pigtail catheters [4,15]. Nakamoto et al. reported a case of intraoperative PTGBD removal resulting in intra-abdominal bleeding [4]. They suggested the following measures to prevent liver injury during PTGBD removal: (1) Straighten the pigtail catheter with the guidewire under fluoroscopy before surgery; (2) Straighten the pigtail catheter with the guidewire during surgery; (3) Intraoperatively, cut the pigtail catheter at the level of the liver bed, remove the straight portion from the body surface, and remove the curved tip with the gallbladder [4]. Koda et al. mentioned the importance of removing the tube after the formation of the fistula, which requires about 20 days, and the specific advantages of removing the catheter after the fistula formation are as follows: (1) It may provide compression hemostasis against vascular injury at the time of puncture; (2) Liver injury may be prevented because the tip of the catheter passes through the fistula at the time of removal; (3) Even if bleeding occurs during removal, it does not result in intra-abdominal bleeding [15]. In this case, the tube was removed without any measures above ten days after insertion, and it may have been possible to prevent bleeding if the above steps had been taken. In addition, in the present case, drainage and hemostasis were first performed from the thoracic cavity for bleeding from the liver via the fistula to stabilize the circulation. Then TAE was performed for intra-abdominal bleeding due to the fistula rupture. Therefore, if the fistula had been severed at the time of laparotomy, bleeding into the thoracic cavity could have been prevented, and the bleeding could have been treated as intra-abdominal bleeding at the time of laparotomy.

## Conclusions

This case report documents a rare but serious complication of massive hemothorax resulting from the removal of a PTAD catheter for bile leak after subtotal cholecystectomy. This case underscores the difficulties of managing acute exacerbations of chronic cholecystitis and the importance of weighing surgical techniques and treatment options carefully. Clinicians should be aware of the potential for this complication and take appropriate steps to prevent it.
